# Microglia, Alzheimer's Disease, and Complement

**DOI:** 10.1155/2012/983640

**Published:** 2012-08-21

**Authors:** Helen Crehan, John Hardy, Jennifer Pocock

**Affiliations:** ^1^Department of Molecular Neurosciences, University College London Institute of Neurology, 1 Wakefield Street, London WC1N 1PJ, UK; ^2^Department of Neuroinflammation, University College London Institute of Neurology, 1 Wakefield Street, London WC1N 1PJ, UK

## Abstract

Microglia, the immune cell of the brain, are implicated in cascades leading to neuronal loss and cognitive decline in Alzheimer's disease (AD). Recent genome-wide association studies have indicated a number of risk factors for the development of late-onset AD. Two of these risk factors are an altered immune response and polymorphisms in complement receptor 1. In view of these findings, we discuss how complement signalling in the AD brain and microglial responses in AD intersect. Dysregulation of the complement cascade, either by changes in receptor expression, enhanced activation of different complement pathways or imbalances between complement factor production and complement cascade inhibitors may all contribute to the involvement of complement in AD. Altered complement signalling may reduce the ability of microglia to phagocytose apoptotic cells and clear amyloid beta peptides, modulate the expression by microglia of complement components and receptors, promote complement factor production by plaque-associated cytokines derived from activated microglia and astrocytes, and disrupt complement inhibitor production. The evidence presented here indicates that microglia in AD are influenced by complement factors to adopt protective or harmful phenotypes and the challenge ahead lies in understanding how this can be manipulated to therapeutic advantage to treat late onset AD.

## 1. Introduction

**Figure 1:**
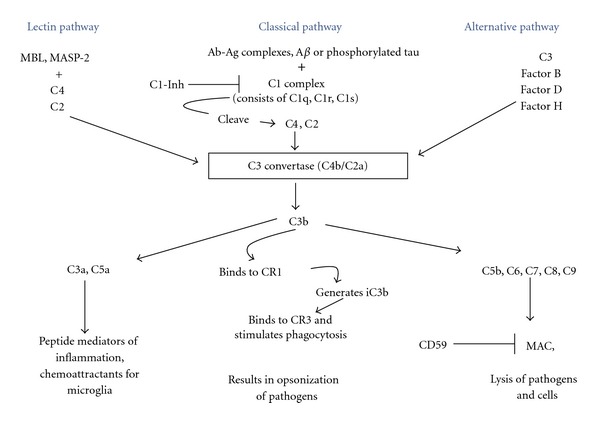
Pathways activating and inhibiting complement. The three complement activation pathways converge at the formation of the enzyme C3 convertase (or C4b/C2a), activation of which leads to the formation of C3b, the ligand of complement receptor 1 (CR1, also known as CD35). Activation of the complement pathway can ultimately lead to the release of inflammatory mediators, opsonisation of pathogens, and the membrane attack complex (MAC). The C1 complex of the classical complement pathway is comprised of C1q, C1r, and C1s. The endogenous complement C1 inhibitor/C1-esterase inhibitor (C1-Inh), which regulates the activation of the C1 complex, is decreased in AD. C5b, C6, C7, C8, and C9 form the MAC complex in the alternative complement activation pathway. CD59, an endogenous regulator of the MAC complex, is decreased in AD whilst C9 may be increased. Levels of Factor H, a regulatory glycoprotein of the alternative complement cascade, may also be perturbed in AD.

The complement system is composed of a series of soluble and membrane-associated proteins present in the blood, which play a role in host defence through the identification, opsonisation, and lysis of pathogenic targets [1–3]. Activation of complement leads to an enzymatic cascade whereby one protein promotes the sequential binding of the following protein [4]. There are three pathways through which complement activation can occur, namely, *classical, lectin,* and *alternative*. Although these pathways depend on different binding molecules for their initiation, they all ultimately lead to the production of complement 3 (C3) convertase which is responsible for the actions of complement [5] (Figure 1). The initiation of the classical pathway involves the binding of C1q, the first protein in the complement cascade, to an antigen-bound antibody complex (IgG or IgM) to either the pathogen surface or to the C-reactive protein bound to the pathogen leading to the generation of the protease C3 convertase through C4 and C2 cleavage [3]. Lectin pathway activation involves carbohydrate binding proteins such as mannose-binding lectin (MBL) or Ficolin binding to carbohydrate elements present on the surface of pathogens, further leading to the production of C3 convertase [6]. The third pathway, the alternative pathway, is different in that there is a constant low level of activation due to the spontaneous hydrolysis of C3 to C3(H_2_O), and this forms C3 convertase through the cleavage of Factor B by Factor D [7]. C3 convertase, the protease formed by all three complement pathways, further binds to the pathogen surface to cleave C3, generating C3b which serves as a ligand for complement receptor 1 (CR1) [8].

The membrane attack complex (MAC) is a macromolecular complex consisting of a number of complement components: C5b, C6, C7, C8, and several C9 molecules whose function is to allow the influx of calcium ions through its ring-like structure resulting in lysis of the target cell [9]. Most cells express protecting/complement regulatory protein/membrane inhibitor of reactive lysis (MIRL or CD59), and this provides protection against MAC as the glycophosphoinositol (GPI-) anchored membrane protein prevents the complete assembly and insertion of the complex into the membrane [10].

The role of complement in the elimination of pathogens by phagocytic recruitment and opsonisation occurs through binding with complement receptors [11, 12]. To date, the family of complement receptors consists of four known types: CR1 (CD35), CR2 (CD21), CR3 (CD11b/CD18), and CR4 (CD11c/CD18). However CR1 has been the best characterized of these since it was discovered almost 60 years ago when it was found that human erythrocytes bound to bacteria treated with complement and a specific antibody [13]. The single-chain glycoprotein that comprises CR1 functions as the receptor to C3b and C4b and as a regulator of complement activation [14]. It is positioned on chromosome 1, band q32, and is composed of a series of short consensus repeats [15]. It is expressed on a number of cells including erythrocytes, B cells, polymorphonuclear leukocytes, monocytes, follicular dendritic cells, and podocytes [16, 17]. Multiple forms of CR1 exist with varying molecular weights ranging from 190 to 260 kDa and the expression of CR1 is under the control of two codominant alleles which code for high (H) and low (L) receptor number, of which the L allele appears to be associated with AD (see review of Crehan et al. [18]). The main role of CR1 on erythrocytes is to remove complement-activating particles and immune complexes from the blood [19].

## 2. Complement Factors and Signalling in Alzheimer's Disease

Studies carried out on mono- and dizygotic twins demonstrate a significant genetic role in the susceptibility to late onset AD (as reviewed by Ertekin-Taner [20]), and until recently APOE was the only known genetic variant to influence the risk of AD development. Recent genomewide association studies (GWASs) have indicated common genetic variations in CLU, CR1, PICALM, ABCA7, BIN1, EPHA1, CD33, CD2AP, and the MSA4 gene cluster as additional risk factors for the development of late-onset AD [21–24]. The genetic variation at CR1 has been confirmed by a meta-analysis with a completely independent data set [25, 26]. CR1 loci demonstrated association with MRI characteristics of AD [27]. The association of the CR1 polymorphism, rs6656401, and cognitive function was measured in 1380 elderly people by Mini Mental State Examination (MMSE), and a cognitive composite score indicated an association between the CR1 polymorphism and poorer performance in the cognitive composite score in males [28]. Recent studies aimed at identifying AD biomarkers have correlated elevated CSF levels of complement factors C3 and C4 in AD patients (with dementia) compared with patients with mild cognitive impairment (MCI) which did not progress to AD [29]. Furthermore CSF levels of CR1 were elevated in patients with MCI progressing to AD and in AD patients, supporting aberrant complement regulation in AD [29].

Classical markers of immune-mediated damage have been identified in AD brains including major histocompatibility complex class I and II positive microglia [30–32], glial cells expressing inflammatory cytokines [33], and the acute phase protein *α*1-antichymotrypsin [34]. Early studies identified complement proteins of the classical pathway, such as factor C1q, in AD brains [35], and subsequent studies established the presence of all of the native complement proteins as well as their activation products C4d, C3d, and MAC in AD brain [2]. However the lack of classical immune complexes led to the search for other complement activators. Rogers et al. [36] demonstrated that, in the absence of antibody, A*β* bound to and activated factor C1q, part of the classical complement cytolytic pathway, and, furthermore, that factors of this complement activation pathway were localised to areas of AD pathology. C1q was subsequently shown to be intimately associated with A*β* plaques [37, 38] as, indeed, have other complement factors such as C3c/d, C4c/d, and C5-9, [38, 39]. The search for antibody-independent activators of the complement pathway continued with the investigation of tau, the major protein component of neurofibrillary tangles. Shen et al. [40] demonstrated complement activation by neurofibrillary tangle material extracted from AD brains and furthermore by human recombinant tau. Whilst most research on complement activation in AD has focussed on the classical pathway, alternative pathway activation also occurs, since the presence of mRNA of the essential alternative pathway element, factor B, has been observed in the frontal cortex of the AD brain [41].

The role of C1q in AD has been experimentally addressed in studies using animal models deficient in the protein. One such study involved the crossing of C1q-deficient mice with a Tg2576 mouse model which exhibits an age-dependent increase in A*β*, dystrophic neuritis, and activated glial cells (microglia and astrocytes) [42]. These authors reported that the number of activated glia surrounding A*β* plaques was lower in the C1q-deficient mice compared with the AD mouse model. In addition there was a reduction in the loss of synaptophysin and MAP2 compared with Tg2576 control mice [42], leading to the conclusion that C1q may have a harmful effect on the integrity of the neuron through initiating an inflammatory response. C1q-deficient mice also exhibited reduced retinal synapse elimination in mouse models of glaucoma, leading to the proposal that C1q mediates synapse loss in other neurodegenerative diseases [43]. Sárvári et al. [44], investigating the effects on hippocampal cells of the C1 complex inhibitor, C1-Inh, showed that inhibition of C1q protected hippocampal cells from A*β*-induced complement lysis. Neurons in the hippocampus and in the cortex are more vulnerable to complement-mediated damage as they are low in the complement inhibitors which usually protect host tissue from complement lysis [45] but conversely are abundant sources of complement [46]. Since these are the two brain areas which correlate with AD pathology, this may explain why analysis of cerebrospinal fluid (CSF) of AD patients indicated significantly lower C1q levels compared with control CSF, and decreased levels of C1q correlate with a diminished cognitive function [47].

## 3. Complement Sources, Inhibitors, and Function within the AD Brain

Complement factors can enter the brain via a compromised blood-brain barrier (BBB). Increasing evidence suggests BBB dysfunction is an early event in AD [48–52]. This may potentiate the triggering of detrimental brain parenchymal signalling cascades by blood components including complement proteins. Additionally the presence of MAC component mRNA and proteins in the AD brain has been reported, suggesting a possible CNS origin of synthesis [53–55]. Whilst neurones are an abundant source of complement proteins [46], the expression of complement protein mRNA has also been observed on murine astrocytes and microglia [56] and in postmortem-derived human CNS microglia [57]. Furthermore, astrocytoma cell lines, astrocytes, and oligodendroglia have been shown to produce complement proteins, indicating glial cells as another potential source of complement factors within the brain [58–61].

Inhibitors of the complement cascade have been shown in biomarker analyses to be reduced in AD. Thus complement C1 inhibitor/C1-esterase inhibitor (C1-Inh), which regulates the activation of complement in the classical activation pathway, has been shown to be reduced in plasma from AD patients [62, 63] and may be the result of the inability of neurons and astrocytes within the AD brain to secrete the active form of C1-Inh [64]. Additionally, deficiencies in the regulation of the alternative pathway of complement activation are also reported in AD. Hence the hippocampus and frontal cortex of AD patients have been shown to display significantly less CD59 expression but more complement factor 9, compared with nondemented control brains [65]. Since CD59 negatively controls MAC assembly, and activity, these data suggest that a deficiency in this control and subsequent damage may contribute to neuronal loss in AD. Factor H is a plasma glycoprotein which regulates the alternative pathway. Factor H is present in A*β* plaques in AD and may bind to CR3 receptors expressed on microglia to generate iC3a [66].

## 4. Microglia and AD

Microglia, resident in normal brain as sentinel cells [67–69], become reactive in AD [70]. In AD, microglia surround damaged or dead cells, clear cellular debris, and predominate around amyloid beta (A*β*) plaques [71]. Microglia proliferate around neurons prior to their loss in murine models of AD [72]. A positron emission tomography (PET) study detecting both activated microglia and an increase in amyloid load correlated the increase in activated microglia with cognitive impairment [73]. Microglia in animal models of AD show reactivity before obvious amyloid plaque deposition [74], indicating an early, “silent” (preclinical and asymptomatic) response of microglia may occur in AD by as yet unconfirmed triggers. These may include amyloid oligomers and hypoperfusion [75, 76], but also complement. Complement activation and activated microglia are early neuropathological events in AD brains [77], and microglial responses show similarity to the peripheral immune system reaction of the macrophage. Activation products of the early complement components C1, C4, and C3 are found within neuritic plaques but there is little evidence of late complement components C7 and C9 or of MAC in the neuropathological lesions in AD brains [78]. This finding leads to the suggestion that in AD the complement system does not act as an inflammatory mediator through MAC formation, but through the actions of the early complement products which fuel the inflammatory responses, leading to neurotoxicity [78].

It is thus increasingly the accepted dogma that inflammation can actively cause neuronal damage and ultimately death of the neuron [79]. Recent data demonstrating that the responsiveness of the innate immune system is higher in offspring with a parental history of late-onset AD indicate heritable traits for AD that are related to inflammatory processes [80]. Furthermore the correlation of higher serum levels of certain acute-phase proteins with cognitive decline or dementia provides additional evidence for the early involvement of inflammation in AD pathogenesis [80]. Microglial reactivity is generally beneficial but the prolonged and progressive nature of the microglial response in AD can promote neurodegeneration. Pathogenic input to microglia, including the enhanced deposition of A*β* peptides, can result in the production of excessive free radicals, proinflammatory cytokines, complement proteins, and glutamate [81–83]. Consequences of the attenuation of inflammation in AD are seen clearly in animal studies. Craft et al. [84] demonstrated that inhibition of glial inflammation in an animal AD model resulted in reduced neurotoxicity. Advanced glycation end product (AGE) accumulation is accelerated in AD as it accumulates on plaques, and AGE-positive neurons and glia both increase with age and dramatically so with AD progression [85]. Activation of the receptor for AGE, (RAGE), on microglia with one of its ligands, such as AGE or A*β*, results in the release of proinflammatory mediators (free radicals and cytokines) [86]. A combination of both these ligands (AGE and A*β*) can lead to an enhanced microglial inflammatory response [87].

## 5. Complement, Phagocytosis, and Microglia

**Figure 2:**
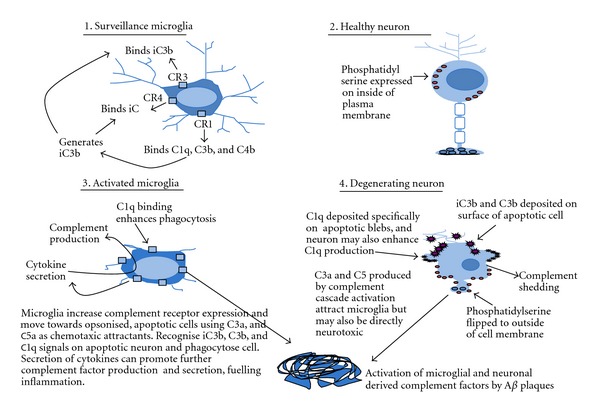
Interactions between microglia and neurons mediated by complement. Surveillance microglia may express low levels of CR1, CR3, and iC3b whilst healthy neurones do not express or produce significant complement. Phosphatidylserine is mainly expressed on the internal surface of the neuronal plasma membrane, preventing it acting as an “eat-me” signal, and complement production by the cell is low. During neuroinflammation and neurodegeneration, activated microglia, responding to the generation of complement factors, increase their expression of complement receptors, produce complement factors, and migrate towards the chemotaxic signals of C3a and C5a. Microglia may exacerbate the secretion of complement factors by secreting cytokines (following exposure to A*β* plaques), which can feed onto astrocytes or form a feedback loop with microglia themselves, promoting glial complement factors secretion. Exposure of secreted complement factors to A*β* plaques can lead to complement activation. Apoptotic neurones become opsonised with iC3b, C3b, and C1q deposition, the latter on apoptotic blebs, and the neurons may also shed additional complement factors. Phosphatidylserine flips to the outside of the plasma membrane where it can potentiate expression of “eat-me” signals by promoting the expression of iC3b on the cell surface [93].

Rat microglia constitutively express C1q and its corresponding receptor CR1 [88, 89] and activated and amoeboid rat microglia, but not ramified microglia, can express CR3, C3 mRNA and shed C3 protein [61, 90]. Human monocytes and macrophages express three known receptors, CR1, CR3, and CR4 that bind complement proteins or their degradation products [91]. CR1 (CD35) binds mainly C3b, C4b, and C1q [16, 89] whereas CR3 (CD11b/CD18) and CR4 (CD11c/CD18) are relatively specific for iC3b [92]. Human macrophages require activation by both alternative and classical complement pathways in order to phagocytose apoptotic cells [93]. Exposure of phosphatidylserine on the apoptotic cell surface is a partial requirement for complement activation and results in coating the apoptotic cell surface with iC3b (Figure 2). The macrophage receptors for iC3b, CR3 (CD11b/CD18), and CR4 (CD11c/CD18) are implicated in this phagocytosis of apoptotic cells and appear more effective compared with other phagocytic receptors, such as scavenger receptors, implicated in clearance [93]. C1q binds directly and specifically to surface blebs of apoptotic cells [94]. Immune complexes coated with C3b and C4b bind to CR1 which leads to their elimination through endocytosis by the CR3 containing phagocytes in the liver [95]. CR1 also functions to regulate complement activation by acting as a cofactor in the Factor-1 mediated cleavage of C3b and C4b [96]. Complement C3 deficiency leads to accelerated A*β* plaque deposition, neurodegeneration, and promotion of a nonphagocytic microglial phenotype in APP transgenic mice [97].

Since complement activation is required for efficient phagocytosis [11] and removal of apoptotic cells within the systemic circulation, early component deficiencies could predispose to systemic autoimmunity by enhanced exposure to and/or aberrant deposition of apoptotic cells [93]. Apoptotic cells promote autoimmunity and defects in the clearance of self-antigens and chronic stimulation of type 1 interferons lead to the systemic autoimmunity seen in C1q deficiency [98]. Null mutations in complement proteins underlie the autoimmune disease systemic lupus erythematosus (SLE), and the severest forms of the disease are those associated with C1q deficiency [99]. Knock-out studies using mice deficient in the complement component, C1q, and patients with systemic lupus erythematosus (SLE) show increased mortality and multiple apoptotic cell bodies and immune deposits, compatible with the hypothesis that C1q deficiency causes autoimmunity by an impaired clearance of apoptotic cells, thought to be a major source of autoantigens in SLE [99, 100]. Further studies have demonstrated the importance of complement in AD. Human-APP transgenic mice expressing the soluble form of the C3 convertase inhibitor Crry (sCrry), (thus hAPP/sCrry mice), showed a 2- to 3-fold higher deposition of A*β* deposits and an accumulation of degenerating neurons compared with the hAPP mice [101]. This suggests that, as in SLE, the impairment of apoptosis in AD and subsequent immune cell responses may fuel disease progression. Changes in complement activation or in CR1 expression [22] might thus lead to a disruption in the clearance of cellular debris and A*β* by microglia. In addition microglial CR3 (CD11b/CD18) is implicated in the phagocytosis of A*β* peptides, acting alongside the low-density lipoprotein receptor-related protein (LRP) [102], and deficiencies in C3bi signalling might thus reduce microglial A*β* phagocytosis. 

 The signalling pathways triggered by complement factors in microglia have attracted modest attention [103–105]. CD88, otherwise known as the complement component C5a receptor 1, plays a role in the calcium signalling required for phagocytosis in microglia ([105], reviewed by [106]). Complement 5a (C5a), a chemotactic agent for macrophages and microglia, transiently activates an outwardly rectifying K^+^ conductance, mediates intracellular calcium mobilisation, and serves to increase microglial motility and to direct these cells by a G-protein-dependent pathway to damaged areas [103, 104]. Recently data suggest that the microglial expression of C5aR/CD88 correlates with A*β* deposition in murine transgenic models of AD, with C5aR/CD88 showing enhanced expression in microglia adjacent to A*β* plaques [107]. Antagonism of microglial C5aR resulted in a significant reduction in pathology in the AD mouse model Tg2576 and reduced hyperphosphorylated tau in 3xTg mice. [107, 108], suggesting a possible therapeutic target for the treatment of AD. Exposure of microglia to complement fragment C3a also induces a calcium response mediated by PTX-sensitive G-proteins [103]. Complement factors have also been shown to increase microglial glutamate transporter GLT-1 expression and promote increased glutamate uptake, without affecting glutamate release [109].

Recent evidence suggests that deletion of C3 convertase regulator complement receptor 1-related protein y (Crry) on microglia results in microglial priming, a microglial state which controversially may precipitate a neurotoxic microglial phenotype and predispose the brain to neurodegeneration [110]. These authors observed that mice that were double-knockout for Crry and either C3 or factor B did not show priming, demonstrating dependence on alternative pathway activation. Colocalization of C3b/iC3b and CR3 implicated the CR3/iC3b interaction in priming, and similar expression patterns were observed in microglia in human multiple sclerosis. In the rodent MS model, EAE was accelerated and exacerbated in Crry-deficient mice and was dependent on complement activation.

Microglia are a source of cytokines, which may, in the AD brain, result in the alteration of complement cascade inhibitors and complement factors. Thus cytokines detected in AD plaques, such as IL-1, IL-6, and TNF-*α*, have been found to differentially stimulate the secretion of C1 sub-components, C1-Inh, C3, and C4 from glial cells including microglia [39]. Microglia constitutively express C1q, whilst the cytokines IL-1*α*, IL-1*β*, TNF*α*, and IL-6 can stimulate the secretion of C1r, C1s, and C3 from microglia, astrocytes, and neuroblastoma cells, and C4 can be secreted in response to IFN*γ* and IL-6, but complement inhibitor C1-Inh is only secreted in response to IFN*γ*. Since this cytokine is not present in A*β* plaques, there is the potential for an imbalance between the generation of complement factors and their inactivation by C1-Inh [39], allowing unregulated activation of complement cascades.

## 6. Complement: A Protective Role in AD and Microglial Responses?

Complement activation in the CNS has been mainly discussed so far with regard to its damaging effects when in fact a number of components of the complement pathway have demonstrated protective effects [111–113]. For example the inflammatory and phagocytotic mediator, C5a, has a protective effect against glutamate neurotoxicity through regulation of the ionotropic Glu2 receptor subunit and protects against neuronal apoptosis [114]. C5a also protected human neuroblastoma cells and normal rodent hippocampal neurons from A*β*-induced neurotoxicity by triggering rapid activation of protein kinase C and activation and nuclear translocation of the NF-kappa B transcription factor [115]. Furthermore, C5a-deficient animals are more susceptible to damage from excitotoxic lesions in the hippocampus [116].

We have previously summarized the detrimental effects of C1q in relation to neurodegenerative diseases. However in contrast, inherited deficiency of this component of the classical complement pathway is associated with systemic lupus erythematosus (SLE) [117, 118] as discussed earlier. C1q mRNA has been reported to be increased in the neurons of patients with AD [119] and other neurodegenerative diseases such as Huntington's disease [120]. Also neuronal C1q synthesis has been demonstrated in the brain of rodent models of ischaemia and excitotoxic insult [121]. Furthermore, an increase in gene expression of C1q has been also demonstrated during normal brain ageing in mice which may be due to an oxidative stress response [122]. This could imply that C1q synthesis may be a response to injury and in fact play a protective role by promoting clearance of apoptotic cells which might otherwise pose an autoimmunity risk [99, 123]. A study in which C1q was incubated with primary neuronal cell cultures revealed a neuroprotective role of this complement factor, when the neurons were exposed to toxic concentrations of A*β* and serum amyloid-P (SAP) [124]. The neuroprotective properties of another complement factor generated from C1q, namely, C3, have also been investigated; a complement C3-deficient amyloid precursor protein (APP) transgenic AD mouse model (APP; C3(−/−)) exhibited accelerated plaque burden in the cortex and hippocampus, increased plasma A*β* levels, and significant hippocampal neuronal loss [97]. Interestingly, the microglia were present in the so-called alternative activation phenotype, displaying significantly increased CD45 immunoreactivity, together with increased brain levels of IL-4 and IL-10 and reduced levels of CD68, F4/80, inducible nitric oxide synthase, and TNF*α* [97]. This would suggest a protective role for C3 in terms of plaque clearance and for its triggering of A*β* phagocytosis by microglia, as shown by Choucair-Jaafar et al. [102] and Fu et al. [125] together with an overall neuroprotection. The findings also reveal that the alternative activation phenotype of microglia in AD may not be a particularly desirous state to aim for as a therapeutic endpoint. Instead it could be argued that some form of microglial response to complement in AD is essential.

## 7. Conclusion

In summary, there is now a substantial body of work implicating alternations in complement signalling in AD. Dysregulation of the complement cascade, either by changes in receptor expression, enhanced activation of different complement pathways or imbalances between complement factor levels, and complement cascade inhibitors may all contribute to the involvement of complement in AD. With regard to microglia and complement, the evidence presented here indicates that microglia can be manipulated by complement factors to adopt protective or harmful phenotypes. Thus, in AD, microglia may be activated by disruptive complement signalling and the presence of A*β* plaques to enhance their secretion of cytokines, which can fuel secretion of further complement factors, leading to a chronic inflammatory response. The task ahead is to unravel further the complex interactions between complement, AD, and microglia as an essential prerequisite to understanding, and manipulating to therapeutic advantage, the role of complement and microglia in AD.
